# Engineered elastin-like polypeptides: An efficient platform for enhanced cancer treatment

**DOI:** 10.3389/fphar.2022.1113079

**Published:** 2023-01-09

**Authors:** Aiguo Jiang, Xinqiang Guan, Lianping He, Xingang Guan

**Affiliations:** ^1^ Department of Respiratory Medicine, Taizhou University Affiliated Wenling Hospital, Taizhou University, Taizhou, China; ^2^ Department of Cardiac Surgery, Lanzhou University Second Hospital, Lanzhou University, Lanzhou, China; ^3^ Department of Basic Medicine, School of Medicine, Taizhou University, Taizhou, China

**Keywords:** elastin-like polypeptide, drug delivery, nanoparticle, inverse transition cycling, cancer therapy

## Abstract

Drug delivery systems (DDSs) have recently gained widespread attention for improving drug loading and delivery efficiency in treating many cancers. Elastin-like polypeptides (ELPs) are synthetic peptides derived from a precursor of elastin (tropoelastin), reserving similar structural and physicochemical properties. ELPs have gained a variety of applications in tissue engineering and cancer therapy due to their excellent biocompatibility, complete degradability, temperature-responsive property, controllable sequence and length, and precisely tuned structure and function. ELPs-based drug delivery systems can improve the pharmacokinetics and biodistribution of therapeutic reagents, leading to enhanced antitumor efficacy. In this review, we summarize the recent application of ELPs in cancer treatment, focusing on the delivery of functional peptides, therapeutic proteins, small molecule drugs, and photosensitizers.

## 1 Introduction

Recent cancer statistics indicate about 19.3 million new cancer cases and 10.0 million deaths caused by cancer worldwide in 2020 (Ferlay et al., 2021). The rising incidence and mortality of cancers promote the urge to develop effective cancer treatments. Surgery, radiotherapy, chemotherapy, targeted therapy, and immunotherapy are common strategies against cancers (Mun et al., 2018). Despite the good outcome of surgery and radiotherapy in treating primary tumors, their applications were greatly limited in treating nonmetastatic tumors (Otake and Goto 2019). Clinical anticancer drugs for chemotherapy, targeted therapy, and immunotherapy have significantly improved the prognosis and survival of cancer patients (Tibau et al., 2018). However, some disadvantages of anticancer drugs, such as poor solubility, inadequate endocytosis, unwanted distribution, and severe side effects, lead to compromised clinical outcomes in some cancers.

Drug delivery systems (DDSs) have shown great potential in improving cancer treatment efficacy through high drug loading capacity, increased cell internalization and tumor distribution, and prolonged circulation time (Dang and Guan 2020; Yang, Wang, and Guan 2022). Many materials, including polymers, lipids, proteins, nucleic acids, and inorganic materials, have been used to construct various delivery platforms to improve the tumor suppression effect (Cheng et al., 2021; Hossen et al., 2019; Patra et al., 2018). Among various DDSs, naturally derived materials-based DDSs attract more and more attention due to their good biocompatibility, excellent biodegradability, and low immunogenicity (Huang and Fu 2010). Elastin, which is widely distributed in mammalian tissues including lung, bladder, blood vessel, skin, and cartilage, helps maintain the elasticity of tissues (Mithieux and Weiss 2005). Elastin-like polypeptides (ELPs), a series of synthetic polymers according to elastin’s amino acid sequence, have shown promising potential in drug delivery for tissue engineering and cancer therapy (Milligan et al., 2022; Shi et al., 2022). This review summarizes the physicochemical properties and the recent applications of ELPs as delivery platforms for cancer treatments.

## 2 Structural and physicochemical properties of ELPs

**FIGURE 1:**
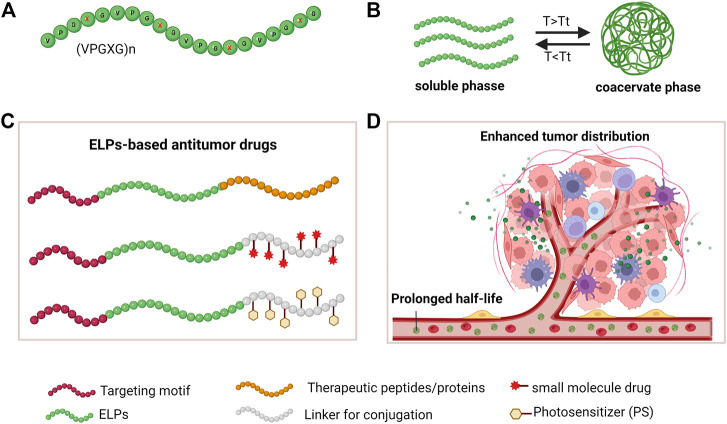
The ELPs properties and strategies of developing ELPs-based antitumor drugs. **(A)** amino acid sequence of ELPs; **(B)** The inverse transition cycling (ITC) property; **(C)** Strategies of developing drug-loaded ELPs by fusion expression and drug conjugation; **(D)** Schematic illustration of ELPs-based drugs in systemically circulating and tumor accumulation.

ELPs, derived from tropoelastin (a precursor of elastin), are synthetic pentapeptide repeats composed of (VPGXG)_n_, while X can be any amino acid except for Pro (Figure 1A). (Megeed, Cappello, and Ghandehari 2002). Due to their naturally derived materials, ELPs have excellent biocompatibility, prolonged circulation, and good elasticity *in vivo*, which make them ideal materials for biomedical application (Suhar et al., 2020). Moreover, precisely adjusting amino acids in the guest residue (X) and repeat numbers (n) make ELPs fine-tuned biomaterials with thermal sensitivity and satisfying mechanical properties for tissue engineering and drug delivery (Ciofani et al., 2014; Gagner, Kim, and Chaikof 2014). In addition, ELPs have a unique temperature-responsive phase-transition property: ELPs undergo from the soluble phase at a lower temperature to ELPs aggregates when the temperature is above the phase transition temperature (Tt), and ELPs aggregates can resolubilize in aqueous solution when the temperature is lower than the Tt (Figure 1B) (Smits et al., 2015). The inverse transition cycling (ITC) property of ELPs has been used to rapidly purify ELPs-fused recombinant proteins with satisfied purity (Floss et al., 2010; Heidari-Japelaghi et al., 2019; Sweet et al., 2021).

ELPs can be fused with bioactive peptides or proteins as fusion proteins by genetic engineering. Functional peptides or proteins are displayed on the surface of protein assemblies and exert their unique functions (Varanko et al., 2020). Moreover, a linker peptide can be fused at the C-terminus of the ELPs sequence for conjugating cytotoxic drugs (McDaniel et al., 2013). In addition, as a block architecture, ELPs can self-assemble into nanoparticles encapsulating drugs by chemical conjugation or physical absorption (Figure 1C) (Rodríguez-Cabello et al., 2016). ELPs-based platforms have demonstrated many advantages for improved delivery efficiency in cancer treatment (Figure 1D) (Shi et al., 2013).

## 3 Drug-loaded ELPs for cancer therapy

**FIGURE 2:**
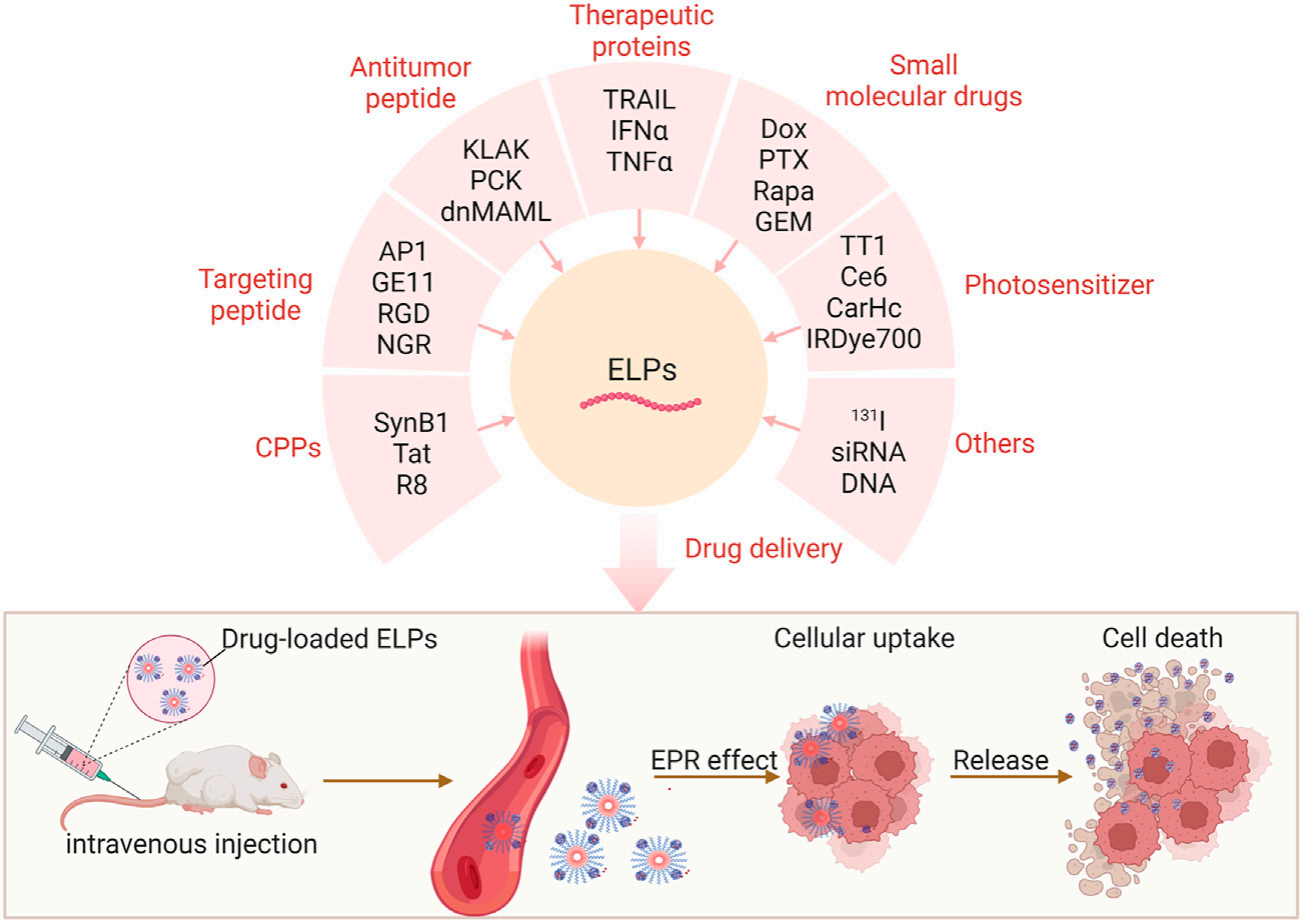
ELPs deliver functional peptides, therapeutic proteins, small molecule drugs, and photosensitizers for enhanced cancer therapy.

ELPs-based DDSs have proven to be an effective strategy to improve the pharmacokinetics of a range of drugs (Milligan et al., 2022). ELPs polymers or nanoparticles could significantly decrease the blood clearance of drugs by reticuloendothelial system and extend the half-time of cargo-loaded (Figure 2), leading to the enhanced antitumor efficacy *in vivo* (Zhaoying et al., 2022).

### 3.1 ELPs delivering tumor-targeting peptides

To endow the ELPs with tumor-selectively binding properties, researchers have decorated many tumor-targeting peptides in ELPs polymer for improved cancer treatment (Matsumoto et al., 2014). Simnick AJ et al. prepared NGR tripeptide-fused ELPs micelles by genetic engineering for tumor vasculature targeting. The self-assembled NGR-ELP micelles showed selective binding with CD13 and improved tumor vascular retention and extravascular accumulation (Simnick et al., 2011). Sarangthem V incorporated multiple AP1 peptides into the ELP polymer for tumor-targeting delivery. The AP1 peptide presented by ELP polymer showed a 10000-fold increase of interleukin 4 receptor (IL-4R) binding affinity compared to the free AP1 peptide (Bhattacharya et al., 2013). Tumor homing peptide F3, a short peptide derived from high-mobility group protein 2, could specifically bind to nucleolin expressed on tumor endothelial cells and tumor cells. F3-ELP nanocarriers delivering doxorubicin showed a 4.2-fold increase in tumor accumulation and a 3.8-fold decreased tumor size compared with ELP nanoparticles without F3 decoration (Hu et al., 2015). Bld-1 peptide, a bladder tumor targeting peptide, has also been fused with ELP(B_5_V_60_) to improve the tumor distribution in bladder tumor xenograft mice (Sarangthem et al., 2018). ELPs fused with gastrin releasing peptide (GRP) could selectively bind with G protein-coupled receptors (GPCRs) and increase the cellular uptake of nanoparticles (Zhang et al., 2016). Cheon SH developed melanoma-targeting ligands-decorated ELPs (M_16_E_108_), which accumulated explicitly in cisplatin-resistant B16F10 melanoma cells and had long retention in tumor tissues (Cheon et al., 2020). Many cell-penetrating peptides (CPP) have been incorporated into ELP polymers to improve tumor cells’ internalization (Massodi, Bidwell Iii, and Raucher 2005). SynB1-functionalized ELP combined with hyperthermia enhances the antitumor effects of Taxol in MDA-MB-231 xenograft models (Ryu, Robinson, and Raucher 2019a). Octa-arginine (R8) incorporation enhanced the penetration of ELP nanoparticles into spheroids generated from human glioblastoma U-87 cells (van Oppen et al., 2019). Therefore, in addition to the high selectivity of tumor-homing peptides with targeting receptors, ELPs decorated with targeting peptides have a prolonged half-life *in vivo*, providing an ideal platform for the efficient delivery of therapeutics agents to the tumors.

### 3.2 ELPs delivering therapeutic peptides or proteins

During the past two decades, therapeutic peptides have attracted considerable attention due to their high selectivity in treating many solid tumors (Fosgerau and Hoffmann 2015; Marqus, Pirogova, and Piva 2017). However, the existence of peptidases and protease *in vivo* significantly limits the application of therapeutic peptides or proteins in cancer treatment (Strohl 2015). A cell cycle inhibitory peptide (p21)-fused with ELPs (p21-ELP1-Bac) demonstrated enhanced cytotoxicity on pancreatic cancer cell lines through cell cycle arrest. ELPs polymers showed synergistic antitumor effects in S2013 xenograft models combined with gemcitabine (Ryu and Raucher 2014). Sarangthem V designed a proapoptotic peptide (KLAKLAK)_2_-loaded ELPs delivery system for treating breast cancer. AP1-ELP-KLAK could significantly enhance tumor localization and suppress tumor growth in breast cancer and melanoma xenografts (Sarangthem et al., 2016). Liu N et al. designed a fluorescent ELPs nanocarrier for targeted delivery of PCK 3145 peptide to epidermal growth factor receptor (EGFR)-overexpressed tumors. The multifunctional ELPs nanoparticles triggered the apoptosis of tumor cells and enhanced antitumor efficacy in the CT26 tumor xenograft model (Liu et al., 2022). A Notch inhibitory peptide (dnMAML) conjugated ELP polymer was developed, showing an enhanced antitumor effect on glioblastoma cells. SynB1-modified complexes inhibited the growth of D54 and U251 cells by inducing apoptosis and cell cycle arrest (Ryu, Robinson, and Raucher 2019b).

As for therapeutic protein delivery, Huang K and his coworkers fabricated tumor-targeting ELPs delivering Tumor necrosis factor-related apoptosis-inducing ligand (TRAIL) protein to colon cancers. ELP-RGD-TRAIL demonstrated a 3-fold increase of apoptosis than without ELP fusion. Single intraperitoneal injection of the RGD-targeted ELPs nanomedicine could inhibit tumor regression in the COLO-205 tumor xenograft model (Huang et al., 2017). ELPs were used to deliver interferon alpha (IFN-α) for cancer treatment. The ELP fused IFN-α showed much higher activity retention than PEGylated IFN-α and IFN-HAS. ELP fusion also significantly prolonged the half-life of IFN-α and excellent antitumor efficacy in ovarian carcinoma xenografts (Hu et al., 2015). Liang P fused IFN-α with ELPs to form a deport injected into the GBM resection cavity. IFN-ELP(V) in the depot dramatically improved pharmacokinetics and biodistribution, and significantly inhibited GBM recurrence, especially combined with temozolomide (TMZ) (Liang et al., 2021). In summary, combining their unique therapeutic mechanisms, ELPs fusion prolonged the half-time of therapeutic peptides or proteins and enhanced their tumor distribution, contributing to enhanced tumor suppression *in vivo*.

### 3.3 ELPs delivering small molecule chemotherapeutics

Due to the prolonged half-time ability, ELPs have been used to improve the delivery efficiency of small-molecule chemotherapeutics for cancer therapy (Bidwell and Gene, 2021). Bhattacharyya J et al. prepared recombinant ELPs containing eight cysteine residues for paclitaxel (PTX) conjugation. PTX-conjugated polypeptides could spontaneously self-assemble into monodisperse nanoparticles, which had twofold increased cell uptake than Abraxane. PTX-loaded nanoparticles near-completely inhibit tumor growth after a single dose in a murine cancer model of human triple-negative breast and prostate cancer (Bhattacharyya et al., 2015). Mie M and his coworkers designed a DNA aptamer conjugated ELPs through a poly-aspartic acid for PTX delivery. PTX-loaded nanoparticles with DNA aptamer showed increased cytotoxicity with MCF-7 breast cancer cells (Mie et al., 2019). Ryu JS conjugated doxorubicin (Dox) to the CPP-ELP polymer using a matrix metalloproteinase (MMP) sensitive linker. CPP-complexed Dox showed significantly increased cell penetration and cell death, even in doxorubicin-resistant breast cancer cells (Ryu, Kratz, and Raucher 2021). Vallejo R prepared docetaxel (DTX) encapsulated, RGD peptide-decorated ELP nanoparticles, suggesting higher cytotoxicity on breast cancer than endothelial cells (Vallejo et al., 2021). Peddi S fused the FKBP12 receptor with ELPs for selective delivery of Rapamycin (Rapa). The Rapa-loaded formulation was internalized by MDA-MB-468 breast cancer cells through macropinocytosis (Peddi, Pan, and MacKay 2018). Avila H prepared an ELPs-based nanocarrier containing rapamycin-binding domains for targeting glucose-regulated protein (csGRP78). The targeted carriers significantly enhanced cellular uptake and reduced mTOR activity by 3-fold compared to free rapamycin (Avila et al., 2022). Ramamurthi D also prepared gemcitabine-conjugated ELPs for ovarian cancer therapy (Ramamurthi et al., 2022). In summary, ELPs delivering chemotherapeutics greatly enhanced their cellular uptake and increased the cytotoxicity against tumors *in vitro* and *in vivo*, suggesting the excellent antitumor effect of the ELPs-based delivery platform.

### 3.4 ELPs delivering photosensitizer

Photodynamic therapy (PDT) has attracted considerable attention in cancer therapy due to precise and non-invasive treatment (Zhang et al., 2020). Photosensitizers (PS) can absorb light to generate cytotoxic reactive oxygen species (ROS) for tumor killing (Shen et al., 2022). PS-loaded ELPs increased tissue permeation and prolonged half-life without loss of ROS production ability (Shi et al., 2022). Pille J prepared llama heavy-chain antibody fragments (VHHs)–decorated ELPs micelles to deliver photosensitizer (IRDye700DX) to EGFR-overexpressed cancers. The PS-loaded micelles demonstrated an EGFR-targeted light-induced tumor cell killing (Pille et al., 2017). Sun M et al. designed a near-infrared absorbing polymer (polypyrrole, PPy)-conjugated PPy-ELP-F3 nanoparticles to treat melanoma. F3-decorated nanoparticles demonstrated higher cellular uptake than nanoparticles without tumor-homing functions in human high-mobility group protein 2 (HMGN2)-overexpressed cancer cells, and they also completely abolish tumors through the combination of photothermal and chemical therapy (Sun et al., 2018). Ibrahimova V and his coworkers conjugated photosensitizer TT1 (a peripherally substituted carboxy-Zn(II)- phthalocyanine derivative) to ELP polymer (M_1_V_3_-40), which could produce singlet oxygen (^1^O_2_) for photodynamic therapy (PDT) (Ibrahimova et al., 2021). Thus, the ELPs platform improved the biocompatibility and tumor accumulation of PS without decreasing their ability to generate ROS for tumor killing, showing a good candidate for the safe and efficient delivery of PS to tumors.

### 3.5 ELPs delivering other drugs

Liu X et al. developed a ^131^I –labeled ELPs for radiotherapy and explored their antitumor effect in rabbits with VX2 liver tumors (Liu et al., 2016). ELPs delivering ^131^I could improve liver function and inhibit tumor growth. Rang-Woon Park group designed ELP nanocarriers containing TAT and AP1 peptides for nucleic acids delivery. TAT-targeted ELPs nanocarriers could selectively deliver siRNA into tumors and significantly downregulate the Luciferase gene expression in the murine breast carcinoma model (Yi et al., 2020). In addition to siRNA, the same nanocarriers could efficiently introduce EGFP plasmids into IL-4R-expressed tumor cells and enhance EGFP expression with low toxicity (Yi et al., 2022).

## 4 Conclusion and perspectives

ELPs have shown great potential in delivering various anticancer drugs for enhanced cancer therapy due to their excellent biocompatibility, complete degradability, temperature-responsive property, controllable sequence and length, and precisely tuned function (Zhaoying et al., 2022). However, many problems remain to be solved for further application of ELPs for cancer therapy. For example, the reported ELPs for drug delivery are prepared using the *Escherichia coli* expression system, which lacks post-translational modification ability. Thus, ELPs may not be suitable for delivering the functional proteins which need sophisticated protein modification. Moreover, the concrete guest residue and peptide length of ELPs need to be optimized for drug delivery to different tumor types. Sarangthem V indicated that increased molecular weight of AP1-ELPs contributes to better tumor penetration and retention in 4T1 tumor-bearing mice (Sarangthem et al., 2020). Kuna M also demonstrated that the increased molecular weight of ELPs had longer plasma half-lives and higher total renal accumulation (Kuna et al., 2018). Due to the high heterogeneity of tumors, the best guest residue and molecular weight of ELPs in delivering antitumor drugs for specific cancer should be investigated in detail. In addition, drug-loaded ELPs nanoparticles hold great promise for next-generation advancement in improving the efficacy of cancer treatment. Therapeutic peptides or proteins could be genetically fused with ELPs for fusion expression, and small molecule drugs or PS could be attached to the backbone or linker peptide of ELPs polymers through post-translational modifications (Milligan et al., 2022). Because of the numerous potential reaction groups on the protein surface, the precise conjugation of antitumor drugs to ELPs remains a significant challenge. Costa SA attached Dox into a nanobody-targeted ELPs nanoparticle by incorporating an unnatural amino acid (p-acetylphenylalanine), providing a promising strategy for targeted conjugation with ELPs polymers (Costa et al., 2018). The biorthogonal drug attachment strategy could be used to develop more and more ELPs-drug conjugates with improved efficacy in the future (Sisila et al., 2022).

In conclusion, ELPs, as flexible and tunable biomaterials, are good candidates for constructing efficient drug delivery platforms for cancer treatment. By improving the pharmacokinetics and tumor distribution, ELPs have enhanced the cytotoxicity of loaded drugs and significantly inhibited tumor growth in many solid tumor xenografts. Despite no approved ELPs-based formulation for cancer therapy, their clinical testing in treating diabetes and heart failure may accelerate the transformation process. The ELPs-based delivery platform will likely bring approved drugs for cancer therapy in the future.
